# A Case of Pneumonia, Accompanied by Transient Albuminuria

**Published:** 1853-03

**Authors:** 


ART. VI.—*A Case of Pneumonia, accompanied by transient albuminuria.*

W. M., a laboring man, about 50 years of age, of a robust constitution, applied for medical advice on the 28th of January. He had been in his usual health till about ten or twelve days previously, when he was exposed to wet and cold during a hard day's work. From that time he had a slight cough, and the appetite was somewhat diminished. Three days afterward he again worked out of doors in the rain, became chilled and wet through, came home with a severe headache, and took to bed. The next day his headache continued severe, with chills, cough, and a sense of oppression in the chest. His appetite disappeared altogether and did not return. He had not much pain in the chest except occasional stitches on the right side, but had much pain in the back, and a general soreness of the limbs. He kept about somewhat, however, every day. On the 28th, as it was pleasant weather, he walked from his house up to the office of his family physician, a distance of three-quarters of a mile; and not finding the doctor at home, returned. Since then he remained in bed. He took two doses of cathartic medicine, which operated freely, and applied a "poor man's plaster" to the right side of the chest; but had no other remedy. He had already lost flesh considerably since the commencement of his illness.

At the time of the first visit, on the 28th, the patient was in bed, but immediately rose, and left his bedroom. He stood up during the examination of his chest, which lasted some twenty minutes, without appearing remarkably fatigued. He showed some emaciation. The countenance was rather pale. The skin, generally, was of a moderate warmth, not moist. A little coolness of skin about the face. Pulse 128, tolerably large and soft. Respiration 38, slightly labored. The tongue was moist, with a thin, whitish coat. There was considerable thirst, but no appetite. He had had some vomiting, after drinking one or two cups of tea. Had had no headache since the first two days of his illness, but complained of feeling "weak." The voice was strong and clear; the cough loose, not very troublesome, not frequent nor painful. Had been rather restless the night previous, though he slept well part of the time. Had taken nothing since morning except tea. Had had one full evacuation from the bowels.

*Examination of Chest.* Has a moderate lateral curvature of the spine, in the dorsal region, toward the right side, and the whole of the right side of the chest is more prominent than the left. Posteriorly the left chest is everywhere resonant on percussion. It rises well in inspiration. Respiratory murmur pure, and the voice of natural resonance throughout, except for a little bronchophony at the root of the lung between the scapula and spine. No râles. The right chest, however, rises during inspiration much less than the left, and the fremitus of the voice, as felt by hand, much greater than on the opposite side. Resonance, on percussion, very slightly, if at all, diminished at any part, but throughout the middle third of the lung there is strong bronchophony and bronchial respiration, extending round quite to the side of the chest. At the lower and lateral parts of this portion of the lung there is a very fine crepitus on full inspiration and often cough; not otherwise. At the lower part of the right back, respiratory murmur pure, and voice natural. Anteriorly,—at the right apex the respiration is somewhat bronchial, but without crepitus. On the left side, respiration pure. On both sides, resonance, on percussion, natural.

He was ordered to keep in bed, and to take cold water for drink, and gruel for food.

℞. Antim. tart. gr. $\frac{1}{8}$.

in solution, every two hours.

*29th.* Reports better. Has less oppression in the chest, and no pain. Took four doses of the tartarized antimony, with only slight nausea and no vomiting. Had one copious loose discharge from the bowels after the third dose, and three more after the fourth; — when the medicine was discontinued. No discharges since. Says he "feels stronger" than yesterday P. M. Is now in bed, lying on back. Countenance as yesterday, except that it is a little less dull. Skin of moderate temperature, not moist. No coolness about face. Pulse 96, same in character as before. Respirations 32, not labored. Tongue as before. Expectoration since yesterday ℥iij. in amount, somewhat viscid, but not excessively so, containing bubbles of air, and of a uniform light-yellowish rusty color.

Examination of the chest gives the same results as yesterday, except that now there is distinct, though moderate, dullness on percussion in the middle third of the right back. The crepitus is very fine, but still has a moist sound. Respiration between spine and scapula tubal. Bronchophony most distinctly pronounced about lower angle of scapula, where the dullness is also most marked. No bronchial sound to-day in respiration in front of right chest. No râle. Resonance, on percussion, also, perfectly good on this point.

The urine since last visit, (17 hours,) is about ℈j. ℥xij. in amount. It is of a natural acid reaction, specific gravity, 1023, and thickly turbid with yellowish-red urate of ammonia; looking like a mixture of weak coffee and milk. On being heated, the urate of ammonia is dissolved, and the urine becomes quite clear, but afterward, on boiling, is again clouded with a deposit of albumen, so as to become nearly as thick as at first. The filtered urine is also clouded by heat and coagulated by nitric acid.

Resume antim. tart. as before, but the medicine to be omitted if purging returns.

30*th.* Reports rather better than yesterday. Oppression less. No pain, except some on right side of chest, on full inspiration. Has taken medicine regularly, without vomiting, and with but little nausea. One loose fœcal discharge, rather less than a pint in amount. Perspiration abundant this morning. Cough same. Pulse 88, of good character. Respirations 32. Expectoration since yesterday, about ℥iij. mostly similar in appearance, but lighter colored, and mixed with some bronchitic sputa. Countenance good, skin of natural temperature.

Examination of the chest gives much the same results as yesterday, except that the dullness on percussion, crepitation, and slight bronchial resonance of the voice have extended to the upper part of the lower third of the right lung posteriorly, and the crepitus in the middle third is rather coarser than before.

The urine, since yesterday, (24 hours,) is a little over ℈ij. in amount, rather less turbid, with urate of ammonia. It is still albuminous, but the albumen less abundant.

Increase antim. tart. to gr. ¼, every two hours.

31*st.* Reports continued improvement. Has taken his medicine regularly. No discharges from bowels. No nausea. Much perspiration. Feels comfortable. Wishes to sit up. Skin warm and moist. Pulse 80. Respiration 26. Tongue the same. Expectoration about ℥iii., not at all colored, but moderately viscid, and quite frothy. Urine, since yesterday, ℈ii. ℥xiv.; still turbid with light yellowish-red urate of ammonia. Albumen continues, also, but is considerably less than yesterday.

Reduce medicine to gr. $\frac{1}{4}$, every three hours.

*Feb.* 1. As well. Countenance a little flushed. No diarrhœa. No nausea. Feels comfortable; only has some uneasiness on right side of chest on cough or long inspiration. The signs, on examination of the chest, were the same, except that the crepitus is rather less abundant, and bronchial respiration and bronchophony less strongly marked. Urine about ℈iii.; still turbid, but deposit lighter colored and less abundant, though it is still copious. Albumen still perceptible, but very slight in amount.

Omit medicine.

2*d.* Has continued to improve. Slept well most of night. One discharge from bowels, nearly natural. Cough very moderate. Sat up half an hour without fatigue. Aspect good. Skin comfortable. Pulse and respiration as yesterday. Tongue a little cleaner. Some appetite. No thirst. Expectoration, ℥iii., nearly all watery and frothy; with only one or two faint streaks of blood.

Examination of chest shows the dullness on percussion, diminished. Crepitus much less abundant and bronchial respiration and bronchophony less pronounced than yesterday, though still rather strong. Respiratory murmur at the *bottom* of the right back has continued pure throughout. Urine a little less than ℈ij, urate of ammonia about the same. No albumen perceptible.

May sit up an hour.

Rice, soda biscuit, and chicken broth for food.

4*th.* As well, or better. One discharge from bowels yesterday. Now countenance natural. Skin in good condition. Pulse 80. Respirations 20. Tongue natural. Sat up two hours yesterday without fatigue. Appetite moderate. No thirst. Cough, and pain in right side, trifling. Expectoration since yesterday, about ℥iss. to ℥ij., perfectly colorless and frothy.

Examination of chest shows the signs of solidification still diminished in extent and intensity. They now occupy a space in the middle third of the posterior part of the lung, about six inches by three. A little crepitus, on forced inspiration, just outside of this space; none elsewhere. Urate of ammonia in urine of yesterday, is diminished in amount, but there is, instead, a considerable quantity of red, crystalline uric acid as a deposit. Urine of to-day, contains some white urate of ammonia, but no uric acid. Very faint indications of albumen still remain.

5*th*. As yesterday. Pulse and respiration the same. Expectoration hardly ℥i. in amount; in appearance as before. Appetite quite moderate. No thirst. Says he has had a disagreeable sensation at the stomach, approaching to nausea, till within a day or two, but none now. Some dullness and bronchophony remain in the posterior middle third of the right chest, but they have diminished since yesterday. No crepitus. Little or no bronchial respiration. Urine about ℥ijj., still with a moderate deposit of white urate of ammonia. Albumen none, or doubtful.

7*th*. Up and dressed. Pulse 84. Feels every way well, except that appetite is not completely restored. Still feels weak. Is somewhat emaciated. Expectoration hardly ℥ss., quite fluid. No physical signs of induration of the lung remain in the right chest, except a slight bronchial tone to voice, at about the centre of the middle third. No crepitus. Respiratory murmur pure. Urine has continued, yesterday and to-day, moderately loaded with the same light-colored deposit as before. No albumen.

9*th*. Has continued improving. Strength increased. Ate a piece of chicken to-day with relish, but the appetite is still only moderate, not craving. No pain. Has coughed only two or three times since yesterday. Countenance well. Pulse 88, soft and full. Patient thinks he has improved more since yesterday than any day yet. Urine since yesterday abundant, rather over ℈iv. by estimate, *perfectly clear and light-colored, without any deposit of urate of ammonia.* No albumen.

11*th*. Better and better. Strength and appetite improving. No morbid symptom. Urine, since yesterday, moderate in amount, clear, and natural in appearance. No deposit or abnormal ingredient. After this time there was no recurrence of the difficulty.

The appearance of albuminuria during the course of acute inflammatory diseases, seems to be no unusual occurrence. It has been observed in pleurisy, pneumonia, pericarditis, bronchitis, inflammation of the brain and spinal cord, hepatitis, &c. In the preceding case its quantity diminished in proportion as the intensity of the pulmonary inflammation subsided. The same, also, was the case with the urate of ammonia deposit, which first became light-colored, and then diminished in quantity, It disappeared, however, more slowly than the albumen, remaining after most of the other morbid symptoms had subsided. At the same time, the patient's appetite returned more slowly than is usual after cases of pneumonia. It will be noticed that the patient experienced the greatest improvement in his sensations on the same day that the morbid deposit disappeared altogether from the urine.

